# A Case Report of a Large Bowel Obstruction and Associated Closed-Loop Small Bowel Obstruction Secondary to Diverticular Disease

**DOI:** 10.7759/cureus.96082

**Published:** 2025-11-04

**Authors:** Kiana Bennett, Jacqueline DeMarco, Marine Bolliet, Paraskevi Orfanou

**Affiliations:** 1 General Surgery, Henry Ford Providence Southfield, Southfield, USA

**Keywords:** anastomotic leak, bowel ischemia, closed loop obstruction, diverticulitis, large bowel resection, small bowel resection

## Abstract

Diverticular disease is one of the most common gastrointestinal pathologies, and its prevalence is rising in areas where diets are low in fiber and high in processed foods containing preservatives and emulsifiers. While this commonly affects the colon, in rare cases, chronic diverticulitis can lead to scarring and fibrosis that impact the small bowel. This case report describes the clinical course of a 56-year-old male with multiple comorbidities who presented to the emergency department for acute abdominal pain with worsening abdominal distention. Imaging revealed extensive intraperitoneal free air, and he was emergently taken for an exploratory laparotomy. Surgery revealed feculent peritonitis, multiple bowel perforations, and a closed-loop small bowel obstruction between the tethered area of pelvic adhesions and a large bowel obstruction extending from the ileocecal valve to the stricture in the distal large bowel. This report highlights the uncommon presentation of simultaneous small and large bowel obstructions originating from related but distinct mechanisms, and emphasizes the importance of understanding how chronic diverticulitis can lead to significant and life-threatening complications. It emphasizes the need to identify causes of bowel ischemia and illustrates the critical role of a multidisciplinary team in managing complex postoperative complications and maintaining nutritional status in patients with significant comorbidities.

## Introduction

Diverticular disease is one of the most common gastrointestinal disorders in both inpatient and outpatient settings [1]. Patients with diverticulosis have a lifetime risk of approximately 1-4% of developing acute diverticulitis [2]. Diverticulitis commonly occurs within the sigmoid colon, presenting with left lower quadrant abdominal pain [3]. Severe cases can lead to perforation, presenting with peritoneal signs such as guarding, rigidity, and rebound tenderness [3]. In some cases, recurrent episodes of diverticulitis can lead to local inflammation, resulting in fibrosis and adhesions [4]. Although diverticulitis commonly affects the colon, in rare cases, these adhesions can impact adjacent structures, including the small bowel.

While prior abdominal surgery is the most likely source of abdominal adhesions, other factors such as trauma, diverticulitis, inflammatory bowel disease, and peritonitis can also contribute [5]. The development of adhesions is known to cause bowel obstructions and is the leading cause of obstructions in the Western world [5]. Bowel obstructions commonly present with nausea, vomiting, and abdominal distention [6]. In the setting of sudden and severe pain, intestinal ischemia and possible perforation should be considered [5]. Ongoing dilation of the intestine eventually causes increased luminal pressure and eventual compromise of the bowel perfusion, which may become irreversible without early intervention [6]. This leads to ischemia, necrosis, and perforation of the bowel [6].

This case report discusses the clinical course of a 56-year-old male with multiple comorbidities who required three open abdominal surgeries and complex postoperative management. His initial surgery revealed a closed-loop small bowel obstruction from pelvic adhesions and concurrent large bowel obstruction from complex diverticulitis. This report aims to highlight a unique presentation of simultaneous large and small bowel obstructions originating from the common etiology of diverticular disease and the importance of optimizing nutritional status and multidisciplinary management of patient care.

## Case presentation

A 56-year-old male presented to the emergency department for acute abdominal pain and increasing abdominal distention over two weeks. He also endorsed decreased appetite and nausea. His past medical history included hypertension, chronic obstructive pulmonary disease (COPD), needing supplemental oxygen, and recurrent diverticulitis. His social history was remarkable for an 80-pack-year smoking history. Upon presentation, his blood pressure (BP) was 103/92 mmHg, heart rate (HR) was 101 bpm, and he was saturating at 89% on room air. Physical exam revealed a distended, firm abdomen with positive rebound tenderness consistent with peritonitis. Pertinent laboratory results revealed lactic acid of 6.1 mmol/L, troponin of 57 ng/L, creatinine of 2.4 mg/dL, blood urea nitrogen (BUN) of 25 mg/dL, and white blood cell (WBC) of 9.16 k/mcL (Table 1).

**Table 1:** Laboratory results from the emergency department

Test name	Patient values	Reference values
Lactic acid, mmol/L	6.1	0.5-2
Troponin, ng/L	57	0-12
Creatinine, mg/dL	2.4	0.6-1.2
Blood urea nitrogen, mg/dL	25	6-20
White blood cell (WBC), k/mcL	9.16	4.0-11

**Figure 1:**
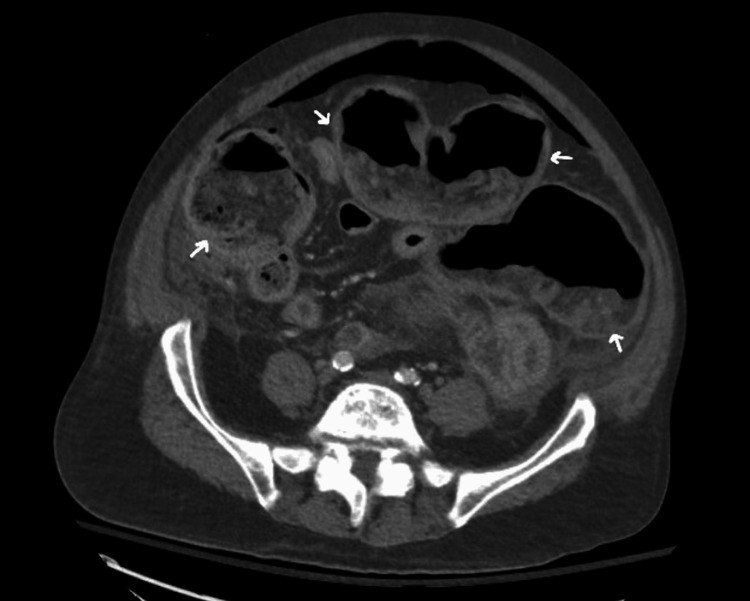
CT abdomen and pelvis with contrast - image 1 White arrows indicate bowel wall thickening consistent with submucosal edema seen in intestinal ischemia. Severe distention of the large bowel with fecal material, fluid and air/gas. There is also free air in the abdominal cavity (pneumoperitoneum) CT: computed tomography

**Figure 2:**
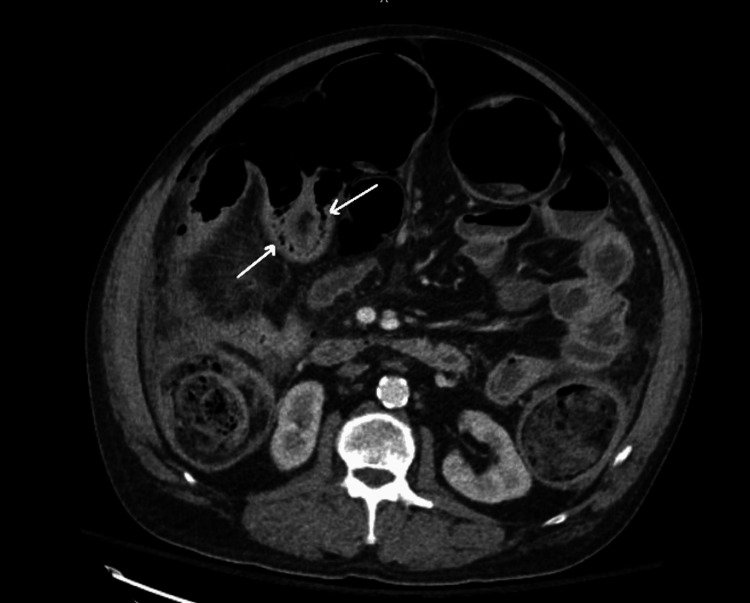
CT abdomen and pelvis with contrast - image 2 White arrows indicate air within the wall of the large bowel (pneumatosis). There is also severe dilation of the large bowel with air fluid levels consistent with an obstruction CT: computed tomography

**Figure 3:**
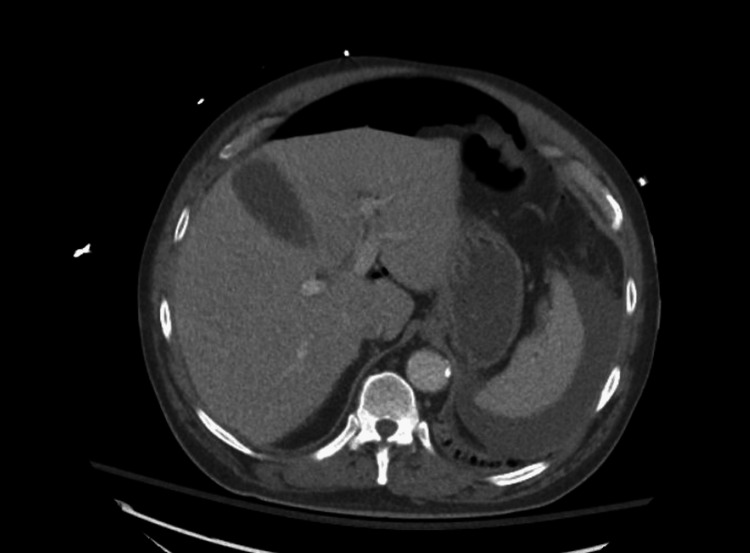
CT abdomen and pelvis with contrast - image 3 Pneumoperitoneum is seen CT: computed tomography

CT of the abdomen and pelvis (CTAP) with contrast revealed thickening of the bowel wall and inflammation consistent with ischemic colitis extending from the rectosigmoid junction to the ileocecal valve (Figure 1). There was associated pneumatosis within affected segments and pneumoperitoneum (Figures 2-3). Based on the patient’s peritoneal examination and imaging findings, it was determined that emergent surgery was required. He was maintained on continuous intravenous fluids, administered preoperative antibiotics, and had a nasogastric tube placed before surgery for decompression.

When entering the peritoneum, stool was noted throughout the abdomen, demonstrating feculent peritonitis. There were perforations noted in the ascending and transverse colon. A small bowel loop was adherent to the pelvis near the rectosigmoid, with significant superficial necrosis at the adhesion site. A small bowel resection was performed, and given the extent of disease and nonviable bowel, a subtotal colectomy was carried out. During the surgery, the patient became hemodynamically unstable, requiring Levophed. As a result, the bowel was left in discontinuity, and an Abthera wound vacuum was placed. He was left intubated and taken down to the ICU.

Postoperatively, his lactate, BUN, and creatinine down-trended, and the patient was extubated and weaned off pressors. He remained under close monitoring in the ICU and required ongoing IV fluid resuscitation. Pathologic examination of the subtotal colectomy and small bowel specimens showed ulceration and necrosis consistent with acute and chronic ischemic colitis. By postoperative day four, he was deemed stable and optimized for a second surgical procedure. The planned second procedure included the creation of an ileostomy, possible additional bowel resection, and abdominal closure. An intraoperative flexible sigmoidoscopy was completed up to 15 cm, but was not advanced further due to a significant stool burden. There were no obvious masses or lesions. Upon re-entering the abdomen, further stool contamination was seen. There were no signs of perforation seen when running the small bowel. A distal rectosigmoid perforation was identified, and a low anterior resection was performed. An end ileostomy was created distal to the prior small bowel resection and anastomosis, as the anastomosis was located too proximally, and placing the ostomy there would have resulted in significant nutritional deficiencies.

**Figure 4:**
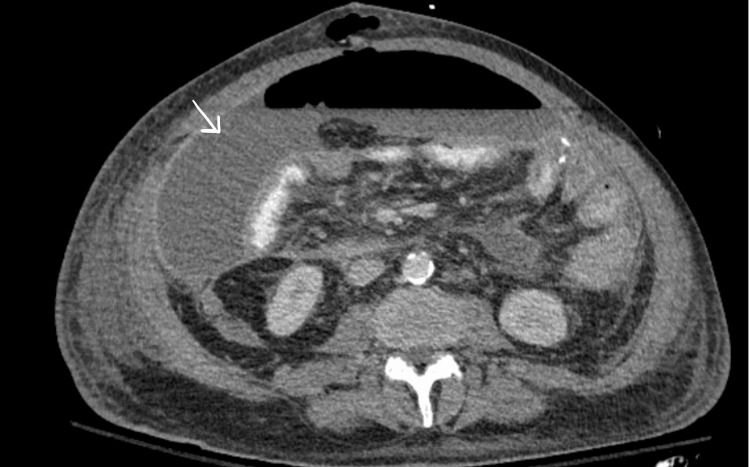
CT abdomen and pelvis with contrast - image 4 The white arrow indicates fluid accumulation. There is also associated intra-abdominal free air consistent with an intra-abdominal abscess CT: computed tomography

The patient was started on total parenteral nutrition (TPN) postoperatively to mitigate worsening electrolyte, nutritional depletions and to aid in wound healing. On postoperative day two from the SS, his ostomy showed good output. Based on his clinical exam, the surgical department advanced his diet as tolerated, but TPN was kept on board to ensure proper nutrition. However, on postoperative day six after the second surgery, his WBC count began to climb, and he was more distended, even though his stoma was functional. A CTAP demonstrated an abdominal fluid collection and increasing free air, possibly representing a large abscess (Figure 4).

Interventional radiology (IR) placed a drain with initial serous output but transitioned to bilious output over 24 hours. Concern for small bowel anastomotic leak (AL) led to an operative take-back. Upon surgical exploration, there was significant fibrinous debris and bile in the anterior abdomen. The entire abdomen was frozen, and the bowel could not be mobilized. The adhesions were too severe to consider lysing due to the high risk of further bowel injury. A 1 cm opening in the left mid abdomen in the small bowel at the staple line was noted. The bowel was too friable, and suture repair of the defect was not possible. A T-tube was placed to create a controlled fistula to the abdominal wall. Two Jackson Pratt (JP) drains were placed in the anterior abdomen. The abdominal fascia was closed, and an incisional wound vacuum was placed. 

**Figure 5:**
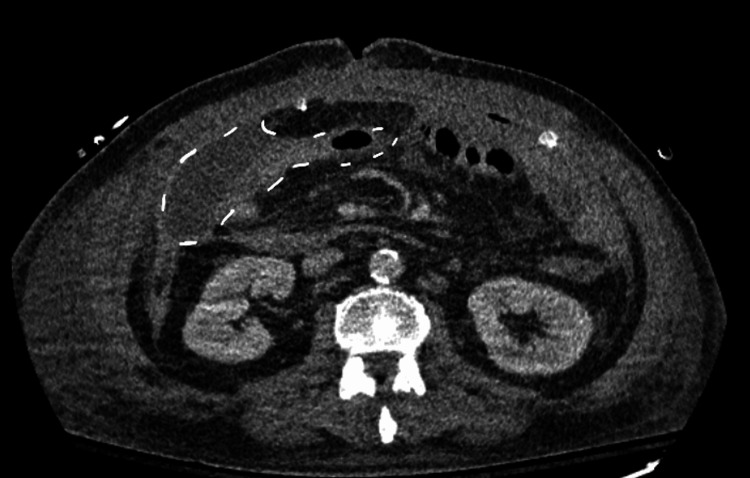
CT abdomen and pelvis with contrast - image 5 White dotted lines indicate remaining fluid collection. This shows improvement compared to Figure 4

On postoperative day two from the third surgery, the T-tube was not draining properly, and an IR T-tube study was done. The study revealed a patent T-tube that remained in the small bowel without signs of contrast extravasation. As the drainage from the T-tube decreased, it was removed by postoperative day five from the third surgery. Minimal drainage from the T-tube indicated resolution of the controlled fistula. A follow-up CTAP was obtained to assess his postoperative progress. This showed that the intra-abdominal wall abscess had decreased in size compared to the image before the third surgery. There was a residual abscess in the right anterolateral abdominal wall that was not being adequately drained by the already present JPs (Figure 5). He underwent another IR drainage. By postoperative day 20 from his third surgery, imaging showed no residual fluid collections, and one of his JP drains was removed. 

Throughout the postoperative recovery after the third procedure, the patient suffered a prerenal acute kidney injury (AKI) that was managed with fluid resuscitation. However, this resulted in pulmonary congestion requiring diuresis. His breathing also required nebulizer treatment due to his underlying COPD. Other specialities such as critical care, nephrology, and nutrition were on board to further aid his postoperative recovery. 

The patient was discharged to inpatient rehabilitation on postoperative day 36 from his initial surgery and postoperative day 23 from his most recent surgery, with one JP drain, an IR drain, and a wound vacuum. The remaining drains were subsequently removed in the outpatient setting following close monitoring and decreased output. The patient continues to do well without any recurrence of complications.

## Discussion

This case highlights how chronic diverticulitis can be an uncommon cause of small bowel obstruction and may be easily overlooked as a potential etiology of the rare concurrent obstruction of both the small and large bowels. It demonstrates the importance of considering diverticular disease as a cause of complex bowel obstruction, even in patients in whom diverticular disease is isolated to distal segments of the large bowel. Delay in identifying such pathology can result in a complex clinical course involving small bowel resection, as experienced by the patient described in this case. 

It is common for patients undergoing emergent gastrointestinal surgery to present with multiple chronic conditions that influence their perioperative risk [7]. This patient had COPD, malnutrition from chronic distention and decreased appetite, and poorly controlled HTN, increasing his risk for postoperative complications such as poor wound healing, infection, and hemodynamic, cardiac, and pulmonary instability [8,9]. Common complications of small bowel resections are anastomotic breakdown or leak, fistula formation, and development of intra-abdominal adhesions.

The incidence of AL or breakdown varies from 1 to 24% based on multiple factors​ [10]. ALs can be seen as extravasation of contrast on enema, fecal material seen in drains or wounds, or fluid collection seen on imaging​ [11]. Acute ALs can be managed operatively or nonoperatively depending on the patient’s condition and radiographic findings. In the absence of clinical symptoms of septic shock, smaller contained ALs can be managed nonoperatively​ [12​]. Small fluid collections less than 3 cm can be managed with antibiotics and bowel rest, while larger leaks greater than 3 cm can be percutaneously drained​ [12].

In the presence of generalized peritonitis or septic shock, patients should undergo surgical intervention​ [13]. There is no established gold standard for managing ALs, as the approach largely depends on the surgeon’s expertise, training, and preference. ALs can either be primarily repaired or require resection. Resection with proximal diversion is the most frequent approach. However, the patient’s quality of life should be taken into account, as ostomy creation can lead to psychosocial challenges, including altered body image and social stigmatization​ [14].

In elective cases, psychiatric counseling should be offered for both temporary and permanent stoma creation. Anastomotic salvage with loop diversion and in combination with anastomotic repair or redo of the anastomosis, can also be considered​ [13​]. In this case, a small bowel AL developed, but repair was complicated by postoperative intra-abdominal adhesions in a frozen abdomen. The adhesions prevented manipulation of the small bowel for salvage or takedown of the anastomosis. Instead, drains were placed near the leak, and a controlled enterocutaneous fistula was created by placing a T-tube in the AL. 

Following the patient’s first emergent surgery, involving a subtotal colectomy and small bowel resection, he remained NPO until a second surgery could be performed. TPN was started immediately postoperatively to prevent nutrition-related complications such as electrolyte depletion and fluid shifts later in the course of his recovery. Additionally, fluid resuscitation and diuresis were balanced in managing both the patient’s AKI and pulmonary congestion postoperatively. He continued to receive breathing treatments to prevent further COPD exacerbation. This highlights the importance of a multidisciplinary approach to patient care, particularly during the postoperative period.

As seen in this patient, intra-abdominal abscesses are a common complication of surgical procedures. They can also arise from gastrointestinal perforation, trauma, or infection from adjacent organs​ [15]. Abdominal abscesses or other collections are commonly managed with percutaneous drainage performed by IR. Collections greater than 3 cm are commonly drained percutaneously​ [16​]. The stability of the patient should also be taken into consideration before deciding on radiologic drainage versus surgical intervention. This further emphasizes a multidisciplinary approach. In the presence of sepsis or multi-organ failure, other treatment options should be explored​ [16​]. Placement of drains enables close follow-up of the abdomino-pelvic collection, observation of the color and consistency, as well as the output of fluid. Over time, the drainage should decrease, indicating resolution of the collection. Persistent drainage could indicate possible fistula formation and would require additional workup and management​ [16]. 

Percutaneous radiological drainage of simple abscesses is a non-invasive procedure with a lower morbidity and mortality compared to open surgical drainage​ [17]. It is a more conservative option for patients who are hemodynamically unstable or not suitable candidates for open surgery, and it generally offers a shorter recovery period compared to laparoscopic surgical treatment​ [17].

## Conclusions

This report discusses a case of chronic diverticulitis leading to the rare occurrence of combined small and large bowel obstruction. It demonstrates how chronic diverticular inflammation can lead to non-surgical related adhesion formation, resulting in eventual obstruction and ischemic bowel necrosis. Additionally, the clinical course highlights the complexities of managing postoperative complications in a patient with multiple comorbidities, including malnutrition, impaired wound healing, pulmonary congestion, acute kidney injury, infection, sepsis, and ALs. It also exhibits the use of a controlled T-tube fistula and image-guided drainage. Ultimately, this report highlights the importance of recognizing chronic diverticular inflammation as a rare cause of small and large obstruction, and the optimization of patient care through multidisciplinary collaboration.
